# Potential Inflammatory Mediators in Pericardial Fluids of Patients With Coronary Artery Diseases and Their Association With Plasma Biomarkers

**DOI:** 10.1111/jcmm.70625

**Published:** 2025-05-28

**Authors:** Reşat Dikme, Mehmet Salih Aydın, Ebru Temiz, İsmail Koyuncu, Mesut Işık

**Affiliations:** ^1^ Department of Perfusion Techniques Program Vocational School of Health Services, Harran University Şanlıurfa Turkey; ^2^ Department of Cardiovascular Surgery, Faculty of Medicine Harran University Şanlıurfa Turkey; ^3^ Department of Medical Biology and Genetics Vocational School of Health Services, Harran University Şanlıurfa Turkey; ^4^ Department of Medical Biochemistry, Faculty of Medicine Harran University Şanlıurfa Turkey; ^5^ Department of Bioengineering, Faculty of Engineering Bilecik Şeyh Edebali University Bilecik Turkey

**Keywords:** biomarkers, coronary artery disease, cytokeratin 18, fetuin A, gene expression, interleukin 33, pericardial fluid

## Abstract

The aim of this study was to determine the gene expression and protein levels of interleukin‐33 (IL‐33), fetuin A and cytokeratin 18 (CK‐18) in the pericardial fluid (PF) and plasma of patients with coronary artery disease (CAD) undergoing coronary artery bypass grafting (CABG). The CAD patients (mean age: 73.4 years) were enrolled. The IL‐33, fetuin A, and CK‐18 protein levels in pericardial fluid (PF) and plasma of patients with CAD were measured by ELISA, while IL‐33 and Fetuin A gene expressions were analysed via quantitative reverse transcription‐PCR (qRT‐PCR). The IL‐33 protein level in PF was significantly higher than plasma (PF: 57.09 ng/L; Plasma: 50.15 ng/L; *p* < 0.05). Similarly, the fetuin A protein levels were significantly elevated in PF compared to plasma (PF: 1060.53 mg/L; Plasma: 725.85 mg/L; *p* < 0.05). However, gene expression levels (ΔCt values) for IL‐33 and fetuin A were significantly higher in plasma than in PF (*p* < 0.05). The CK‐18 protein levels were comparable between plasma and PF (*p* > 0.05). Strong positive correlations were observed between CK‐18 and IL‐33 (*r* = 0.127, *p* < 0.001) and between CK‐18 and fetuin A (*r* = 0.096, *p* < 0.001) in PF. The IL‐33, fetuin A, and CK‐18 levels in PF are predicted to have the potential to be used as a source of biomarkers for CAD. Although the collection of PF samples requires an invasive procedure, the proximity of PF to the heart tissue makes it a valuable source for understanding cardiac pathophysiology. These findings highlight the potential diagnostic and therapeutic utility of PF biomarkers in patients with CAD.

## 1 Introduction

Coronary artery disease (CAD) remains the leading cause of mortality and morbidity worldwide, primarily driven by chronic inflammation and atherosclerosis. Inflammatory biomarkers have been identified as valuable indicators of clinical outcomes in CAD, with studies revealing significant differences in oxidative stress markers and metabolic gene expression, which are useful in diagnosing atherosclerosis [1, 2, 3, 4]. Effective diagnostic studies and biomarker monitoring are crucial for this disease. Biomarkers of oxidative stress and metabolic dysfunction have been widely investigated to aid in the early detection and management of CAD [1, 3, 4]. However, conventional circulating biomarkers often fail to fully reflect localised cardiac‐specific pathophysiological processes [2].

The potential biomarkers such as interleukin 33 (IL‐33), fetuin A, and cytokeratin 18 (CK‐18) are critical for identifying new diagnostic and therapeutic strategies for CAD, given their roles in inflammation, tissue repair, and disease progression. Interleukin‐33 (IL‐33), fetuin A, and CK‐18 have emerged as significant mediators of inflammatory and apoptotic pathways in CAD [5, 6, 7, 8, 9, 10, 11, 12, 13]. IL‐33, a member of the IL‐1 cytokine family, has been reported to attenuate myocardial fibrosis and apoptosis through modulation of anti‐apoptotic proteins [7, 8, 9]. However, IL‐33 also displays complex roles, exhibiting both protective and pro‐inflammatory effects in cardiovascular diseases. Fetuin A has been recognised for its dual role, inhibiting vascular calcification and exerting anti‐inflammatory effects, while paradoxically promoting insulin resistance and potentially contributing to CAD development [10, 11]. CK‐18, primarily expressed in vascular smooth muscle cells, is implicated in cytoskeletal changes during atherosclerosis progression and serves as a marker of apoptosis [12, 13].

Although many biomarkers are used to determine the risk of CAD, they are often insufficient to fully assess the actual risk, as the complex pathophysiology of CAD requires the simultaneous evaluation of multiple biomarkers rather than relying on a single parameter [5, 13]. Thus, a more comprehensive approach is necessary to capture the full spectrum of disease‐related changes. In this context, a biopsy sample could offer more accurate information, and pericardial fluid (PF), which is directly associated with heart tissue, can be examined to gain deeper insights into CAD pathophysiology.

While blood‐based biomarkers provide important systemic information, there remains a significant knowledge gap regarding biomarker expression in PF—a biofluid in direct contact with cardiac tissues [14, 15]. PF reflects myocardial interstitial components and may offer insights into localised inflammatory and apoptotic processes associated with CAD [14, 15]. Various biochemical markers related to myocardial injury have also been detected in PF, highlighting its potential as a source for cardiac‐specific diagnostic information [15]. However, comprehensive evaluations comparing both gene expression and protein levels of IL‐33, fetuin A, and CK‐18 in PF and plasma have not yet been performed. In cardiovascular diseases, which remain the leading cause of death worldwide, PF analysis alongside blood and heart tissue examinations can provide crucial insights into underlying pathophysiological mechanisms [16].

Studies exploring the relationship between protein and gene expression levels of these biomarkers in PF and plasma of patients with CAD are limited. Thus, the aim of this study was to simultaneously investigate the protein and gene expression levels of IL‐33, Fetuin A, and CK‐18 in PF and plasma of patients with coronary artery disease undergoing coronary artery bypass grafting (CABG), as well as their relationship with each other, thereby providing new insights into potential cardiac‐specific biomarkers for diagnosis and therapeutic intervention.

## 2 Methods

### 2.1 Study Population and Processes

Forty patients who had coronary artery bypass surgery with cardiopulmonary bypass (CPB) method were included in this study. The study that was conducted according to provisions of the Helsinki Declaration was approved by Harran University, Faculty of Medicine, Ethics Committee (decision number 05.01.2017–17/01/23). All patients provided written informed consent to participate in this study. For patients who were unable to provide consent themselves, consent was obtained from their legally authorised representatives. PF and venous blood samples were obtained. In this study, a control group was not included due to the limited number of patients undergoing thoracotomy for non‐cardiac‐related reasons in the region where the study was conducted and the ethical challenges associated with obtaining PF samples from such patients. While including a control group could provide additional comparative insights, the invasive nature of PF sampling further limits its feasibility. Instead, this study focused on analysing intra‐patient variations in plasma and PF biomarkers, which reflect the local cardiac environment in CAD patients. The 40 volunteers with CAD (21 men and 19 women, average age 73.4 years) were included in the study.

### 2.2 PF and Blood Plasma Sampling

PF and blood sampling were performed simultaneously during the surgical procedure to ensure consistency in the physiological conditions of the patients. In the scope of the CPB procedure, after median sternotomy was performed in patients who underwent coronary bypass surgery, the pericardium was dissected and the PF was aspirated with a sterile syringe. The aspirated PF (2–25 mL) was then taken into sterile tubes without anticoagulant. Then, the PF was centrifuged 2 times at +4°C for 5 min at 5000 rpm and the supernatant was transferred to an RNase‐free tube (Eppendorf tube) and stored at −80°C. Venous blood samples were taken from the patients and transferred to heparinized sterile tubes. Then, the tubes were centrifuged at 5000 rpm for 5 min and the supernatant was transferred to an RNase‐free tube (Eppendorf tube) and stored at −80°C.

### 2.3 Gene Expressions of IL 33 and Fetuin A

#### 2.3.1 Total RNA (mRNA) Isolation

Qiagen miRNeasy Serum/Plasma Kit (Qiagen GmbH, Hilden, Germany, Cat No./ID: 217184) was used to isolate total mRNA in the PF and plasma according to the manufacturers' protocols.

#### 2.3.2 cDNA Synthesis

RT^2^ First strand kit (RT^2^ HT First Strand Kit, Qiagen, GmbH, Hilden, Germany, Cat No./ID: 330411) was used to synthesise cDNA from total mRNA according to the manufacturer's protocol. Determination of quality and amount of the obtained cDNA samples was performed with Thermo Scientific Multiskan Spectrophotometer System (Waltham, Massachusetts, US) before gene expression studies.

#### 2.3.3 mRNA‐cDNA Preamplification

The ‘mRNA‐cDNA preamplification’ step involves amplifying specific target genes from synthesised cDNA to enhance the sensitivity and reliability of subsequent qRT‐PCR analysis. This step was performed using a primer pool designed to target IL‐33, Fetuin A, and the housekeeping gene Beta‐Actin, ensuring accurate quantification even for low‐abundance mRNA transcripts. By preamplifying these target regions, we minimised the potential loss of signal during the qRT‐PCR process.

Amplification, 40 μL primer (20 μL F (10 pmol/μL) + 20 μL R (10 pmol/μL)) was used for the primer pool. 210 μL of water was added per primer, and the relevant content is summarised in Table 1. The reaction was carried out at 25.5 μL by adding 5 μL of cDNA to the total mix.

**TABLE 1:** Primer pool mix for gene expression.

One sample	
5x miscript master mix	12.5 μL
Taq polymerase	0.5 μL (250 unit)
Primer pool	7.5 pmol/μL
Total mix	20.5 μL

#### 2.3.4 Primer Desing

IL 33 and Fetuin A primers (metabion international AG/metabion GmbH, Germany) and Beta Actin as housekeeping were used. IL 33 and Fetuin A primers were designed by using ‘gene’ interface in the NCBI database (http://www.ncbi.nlm.nih.gov/tools/primerblast/), and the forward and reverse primer sequences of these primers are shown in Table 2.

**TABLE 2:** Il 33, Fetuin A and Beta Actin forward and reverse primers.

	Forward primer	Reverse primer
IL 33	TTGGGTGTTGGGGTATTTTGC	GCTCTTTCCTCAGTGGCCTT
Fetuin A	CAACCGAACTGCGATGATCC	TTGGAACACCATGCAGGTCAC
Beta Actin	CGTACCACAGGCATTGTGATG	TTTGATGTCACGCACGATTTC

#### 2.3.5 PCR Protocol for IL 33 and Fetuin A Genes

Rotor Gene 6000 Real‐Time PCR Machine (Qiagen GmbH, Hilden, Germany) and QuantiTect SYBR Green PCR Kit (Qiagen GmbH, Hilden, Germany, Cat No./ID: 204143) were used for qRT‐PCR reaction. The components used in qRT‐PCR are summarised in Table 3.

**TABLE 3:** qRT‐PCR protocol for IL 33 and Fetuin A expression level.

Components	Volume
RT^2^ SYBR Green Mastermix	5 μL
F Primer (10 pmol/μL)	0.3 μL
R Primer (10 pmol/μL)	0.3 μL
RNaz/DNaz‐ free water	3.9 μL
cDNA (50 ng/μL)	0.5 μL
Total	10 μL

The prepared premix was distributed in tubes as 9.5 μL; 0.5 μL of cDNA was added to tubes containing the PCR premix, and the study was performed under the qRT‐PCR conditions summarised in Table 5. The experiment comprises the steps of the reaction mixture incubated 2 min at 50°C for the initial step, 10 min at 95°C for deactivation, and subsequently 40 cycles of 15 s at 95°C for denaturation and 1 min at 60°C for annealing and extension.

### 2.4 Quantitation of IL 33, Fetuin A and CK 18 Level With ELISA

IL 33, Fetuin A, and CK 18 protein levels in plasma and PF were determined by the ELISA method by applying the relevant kit protocols. Human (IL 33) ELISA Kit (Sunred Shanghai Sunred Biological Technology Co. Ltd., Cat. No: 201‐12‐0045) was used to detect IL 33 protein level. Human Fetuin A ELISA Kit (Sunred Shanghai Sunred Biological Technology Co. Ltd., Cat. No: 201‐12‐1387) was used to detect Fetuin A protein level. Human (CK 18) ELISA Kit (Sunred Shanghai Sunred Biological Technology Co. Ltd., Cat. No: 201‐12‐1716) was used to detect CK 18 protein level.

### 2.5 Statistical Analysis

This study employed statistical analysis using the SPSS 28.0 package program (US) and the R programming language to assess the relationship between variables. Pearson correlation analysis was utilised to evaluate the association between variables. The results were exhibited as mean ± standard error of the mean (95% confidence intervals). In comparison of normally distributed continuous variables between two independent groups, the independent samples *t* test was used. To explain the variance in continuous variables by independent continuous variables, multiple regression analysis was performed. Differences between data sets were considered statistically significant when the *p*‐value was less than 0.05.

## 3 Results

**FIGURE 1:**
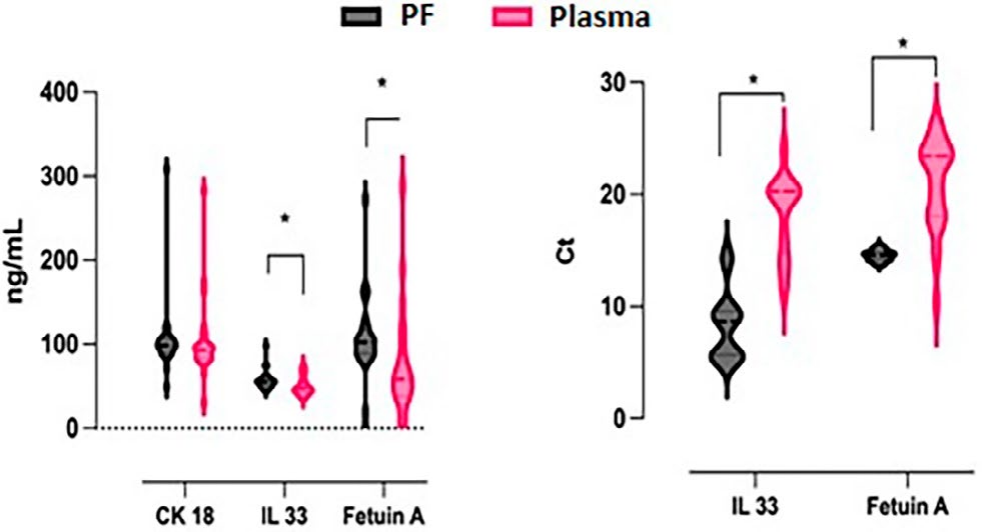
A correlation heatmap based on assessing the relationship between variables such as CK 18, IL33, Fetuin A in the plasma and PF.

Statistical analysis findings are summarised as hypothesis, regression and correlation tests in the following tables. CK 18, IL33, Fetuin A, IL33 ∆Ct ve Fetuin A ∆Ct variables was compared between independent groups, the protein and gene expression levels (indicated as ΔCt, which represents the difference in threshold cycle (Ct) values) were summarised in Table S1. The IL33 ΔCt and Fetuin A ΔCt variables represent the normalised gene expression levels, calculated as the difference between the Ct value of the target gene (IL‐33 or Fetuin A) and the housekeeping gene Beta‐Actin. This approach ensures accurate comparison of gene expression levels across samples. The CK 18, IL33, Fetuin A, IL33 ∆Ct and Fetuin A ∆Ct variables were compared between independent groups. According to the analysis results, there was no significant difference between PF and plasma CK 18 protein level measurements (*t* = 0.176, *p* > 0.05, *d* = 0.048). The IL‐33 protein level in PF was significantly higher than its corresponding level in plasma (*t* = 2.362, *p* < 0.05, *d* = 0.643). This indicates a localised increase in IL‐33 protein concentration in PF compared to systemic circulation. Fetuin A protein level in PF was significantly increased compared to Fetuin A's level in plasma (*t* = 2.262, *p* < 0.05, *d* = 0.607). The IL‐33 gene expression level in plasma was significantly higher than its expression in PF (*t* = −10.931, *p* < 0.05, *d* = −0.975). The higher IL‐33 gene expression in plasma but increased protein levels in PF can be explained by several factors. Post‐translational modifications and protein stability may lead to longer IL‐33 retention in PF. Additionally, IL‐33 might be locally produced in the pericardial environment or actively transported from plasma to PF. The protein may also accumulate in PF due to cellular injury or apoptosis occurring in cardiac tissues. These factors contribute to the observed differences between gene expression and protein levels [17, 18] in plasma and PF. This suggests that the increase in IL‐33 mRNA expression may be more actively transcribed in circulating blood than in PF, despite the higher protein concentration in PF. Plasma Fetuin A gene expression was found to be significantly enhanced compared to the Fetuin A's gene expression in the PF (*t* = −8.387, *p* < 0.05, *d* = −0.823). While the protein levels of CK‐18, IL‐33, and Fetuin A were significantly higher in PF compared to plasma, the gene expression levels of IL‐33 and Fetuin A were found to be higher in plasma. This suggests that these biomarkers may be locally concentrated or regulated in the cardiac environment, making PF a valuable source for heart‐specific biomarker analysis. Protein and gene expression levels for potential biomarkers in the groups are summarised in Figure 1.

**FIGURE 2:**
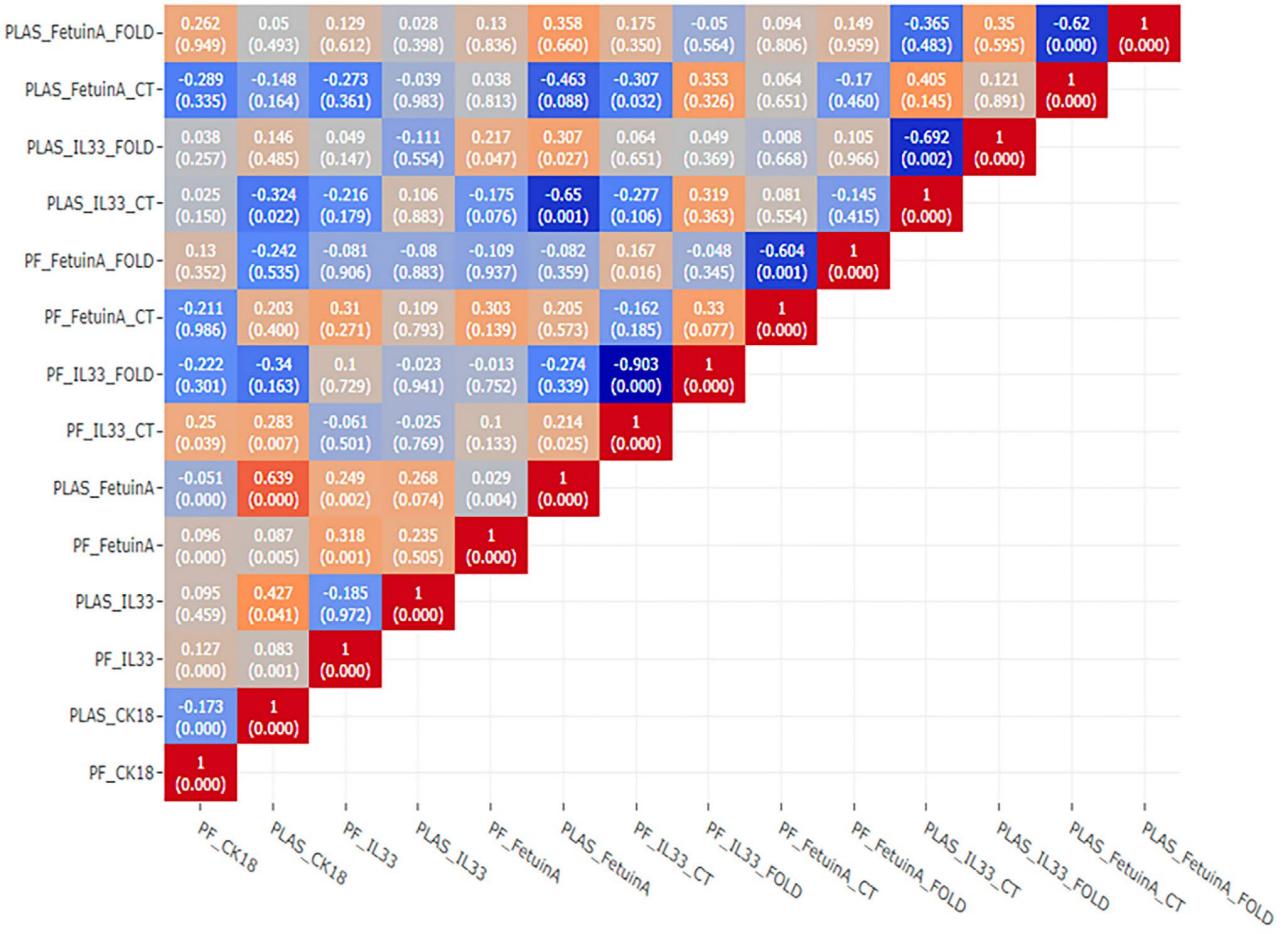
Correlations between protein levels and gene expressions of CK 18, IL33 and Fetuin A.

Heatmap graph showing the detailed correlation of plasma and PF (CK 18, IL33, Fetuin A, IL33 ∆Ct and Fetuin A ∆Ct, etc.) variables with each other is presented in Figure 2. According to the results of Pearson correlation analysis, there is a significant positive correlation between CK 18 and IL 33 protein levels in PF (*r* = 0.127, *p* < 0.001). At the same time, PF_CK 18 shows a positive correlation with PF_Fetuin A (*r* = 0.096, *p* < 0.001) and a negative correlation with plasma CK 18 (PLAS_CK 18, *r* = −0.173, *p* < 0.001) and PLAS_Fetuin A (*r* = −0.051, *p* < 0.001). PLAS_CK 18 shows a positive correlation with PF_IL33 (*r* = 0.083, *p* < 0.001), PF_Fetuin A (*r* = 0.087, *p* < 0.01), PLAS_Fetuin A (*r* = 0.639, *p* < 0.001) and PF_IL33_CT (*r* = 0.283, *p* < 0.01). PF_IL33 shows a positive correlation with PF_Fetuin A (*r* = 0.318, *p* < 0.001) and PLAS_Fetuin A (*r* = 0.249, *p* < 0.01). The negative correlations between IL 33 and Fetuin A ∆Ct and protein levels are thought to be caused by the post‐translational regulation mechanisms. Multivariate regression analysis was performed to investigate the cause of the positive correlation between CK 18 and anti‐apoptotic Fetuin A protein levels.

A multivariate linear regression analysis was performed to predict the CK 18 variable using the IL33 and Fetuin A independent variables (Table 4). As a result of the analysis, it was found that a significant regression model (F(4.49) = 5.135, *p* < 0.05, and 23.8%) of the variance in the dependent variable (*R* ^2^_adjusted_ = 0.238) was explained by the independent variables. Accordingly, the independent variable IL33 predicted the dependent variable positively and significantly (*β* = 0.564, t(49) = 4.031, *p* < 0.001, pr^2^ = 0.25). The independent variable Fetuin A predicted the dependent variable negatively and not statistically significantly (*β* = −0.083, t(49) = −0.560, *p* > 0.05, pr^2^ = 0.06). The independent variable IL33 ∆Ct predicted the dependent variable positively and not statistically significantly (*β* = 0.277, t(49) = 1.623, *p* > 0.05, pr^2^ = 0.051).

**TABLE 4:** Regression of CK 18 by IL33 and Fetuin A protein levels and gene expression.

Model		Unstandardized coefficients		Standardised coefficients	*t*	Sig.	Correlations			Collinearity statistics	
		B	Std. Error	Beta			Zero‐order	Partial	Part	Tolerance	VIF
	(Constant)	−223.683	388.659		−0.576	0.568					
	IL33 ng/L	21.842	5.419	0.564	4.031	< 0.001	0.478	0.499	0.483	0.735	1.360
	Fetuin A mg/L	−0.064	0.115	−0.083	−0.560	0.578	0.129	−0.080	−0.067	0.649	1.540
	IL33 ∆Ct	20.064	12.365	0.277	1.623	0.111	0.137	0.226	0.195	0.494	2.025
	Fetuin A ∆Ct	−6.781	17.518	−0.067	−0.387	0.700	0.018	−0.055	−0.046	0.480	2.082

Independent variable Fetuin A ∆Ct predicted the dependent variable negatively and was not statistically significant (*β* = −0.067, t(49) = −0.387, *p* > 0.05, pr^2^ = 0.030).

Plasma protein levels of CK 18, IL33 and Fetuin A recorded in the study were compared with the population mean of healthy individuals (Table 5). According to the results of the analysis, the CK 18 level of patients with bypass increased significantly compared to the mean of healthy individuals in the same population (*t* = 11.841, *p* < 0.001, *d* = 0.729). The IL33 level of bypassed patients was significantly increased compared to the mean IL33 of healthy individuals in the same population (*t* = 20.151, *p* < 0.001, *d* = 0.878). Fetuin A level of bypassed patients increased significantly compared to the mean of healthy individuals in the same population (*t* = 3.241, *p* < 0.001, *d* = 0.624.). This increase may have high clinical impact.

**TABLE 5:** Comparison of the observed markers with the test value.

	Mean	Std. Deviation	Test value	*t*	*d*	*p*
CK 18 ng/mL	1025.3181	448.55823	301	11.841	0.729	0.001
IL33 ng/L	50.1556	10.79274	8.3	20.151	0.878	0.001
Fetuin A mg/L	725.8565	599.42095	352	3.241	0.624	0.002

## 4 Discussion

In this study, we comprehensively evaluated the gene expression and protein levels of IL‐33, fetuin A, and CK‐18 in pericardial fluid (PF) and plasma of patients with CAD undergoing CABG. Our findings revealed that protein levels of IL‐33 and fetuin A were significantly higher in PF compared to plasma (*p* < 0.05), whereas their gene expression levels were significantly higher in plasma. CK‐18 protein levels, although elevated, showed no significant difference between PF and plasma (*p* < 0.05). Positive correlations were observed between CK‐18 and IL‐33 and fetuin A levels in PF (*r* = 0.127, *p* < 0.001). These results highlight the potential role of PF biomarkers in reflecting cardiac‐specific pathological processes and suggest their applicability in diagnostic and therapeutic approaches for CAD.

IL 33, Fetuin A and CK 18 in PF are new biomarkers that are effective in the diagnosis, prognosis and treatment of CAD. However, IL 33 has complex and conflicting roles in the pathophysiology of CAD. IL 33, which has a number of effects on endothelial and immune cells, can both promote inflammation and atherosclerosis and inhibit plaque development and progression. It has not been determined why IL 33 plays pro‐inflammatory or anti‐inflammatory roles [19, 20].

In a study conducted in 2018, IL 33 serum concentration was measured in a healthy control group (61.85 pg/mL), AMI (103.33 pg/mL), stable angina patients (157.60 pg/mL) and unstable angina patients (122.21 pg/mL). According to these results, it was determined that serum concentration of IL 33 in patients with ischemic heart disease was higher than that in healthy control groups [21]. Xin Tu et al. (2013) found plasma IL 33 concentration as 233.67 pg/mL in 51.6% of the patients with CAD [7]. In our study, the IL 33 level was found to be increased in PF compared to plasma, which is inconsistent with the previous studies. This contradiction suggests that to standardise the IL 33 level for CAD, more studies are required to enlighten temporal changes in the pathophysiology of CAD.

According to the gene expression results as potential biomarkers, IL 33 was determined to be higher in plasma than PF. This proposes that PF can reflect physiological and biochemical changes in the heart, and thus its potential for diagnostic and therapeutic purposes. It has been determined based on the obtained data that Fetuin A has both positive and negative effects on CAD. While Fetuin A's ability to inhibit coronary artery calcification and have anti‐inflammatory effects is positive, its ability to induce CAD development by increasing insulin resistance at the tyrosine kinase level is negative. Therefore, the effect of Fetuin A on CAD depends on the combination of several factors in different situations or conditions [10].

Chen et al. found that Fetuin A was associated with increased mortality in patients with CAD [10]. In the study, patients were divided into three groups according to their plasma Fetuin A levels (low level ≤ 0.64 g/L; moderate = 0.65–0.86 g/L; high level = 0.87–2.17 g/L), and young patients were found to have higher plasma Fetuin A levels (0.87–2.17 g/L). In addition, higher Fetuin A levels were correlated with less narrowing of the coronary artery [10]. In another study, the serum concentration of Fetuin A was found to be between 400 mg/L and 1000 mg/L [22]. In our ELISA study, while the Fetuin A level was 1060.53 mg/L (1.06 g/L) in PF, it was 725.85 mg/L (0.72 g/L) in plasma. When we compared the Fetuin A levels we obtained with the previous studies, we saw that the Fetuin A level was high in the PF and normal in the plasma of our patients. In our gene expression study, the Fetuin A was recorded as 14.55 in PF and 21.02 in plasma, showing that Fetuin A was relatively higher in PF. Because a low Fetuin A level is correlated with a high risk of CAD, our findings suggest that Fetuin A probably decreases mortality risk by inhibiting the calcification in the cells.

Playing a role in the cell death pathway, CK 18 is expressed in vascular VSMCs associated with atherosclerotic plaques [23]. According to the study conducted by Qian et al. (2019), the increased serum level of CK 18 was accepted as an independent indicator of cardiometabolic disorders [12, 24]. In the study, the CK 18 level was found to be 83.85 U/L in the control group and 197.36 U/L in people with cardiometabolic disease. In another study conducted in 2017, while the total CK 18 was found to be 262.9 U/L in the patients with cardiometabolic disorders, it was found to be 158.7 U/L in the control group [23]. These studies suggest that the CK 18 level is increased in patients with cardiovascular problems. The strong correlation between serum CK 18 levels and the risk of cardiometabolic disorders can be explained by many factors [12]. In our study, the CK 18 level was measured in both plasma and PF. According to the ELISA results, the mean CK 18 level was found to be 1046.42 ng/mL in PF; it was 1025.31 ng/mL in plasma. The increased CK 18 level in both PF and plasma gives us a clue that necrosis or apoptosis has been triggered in CAD patients. The study reveals a statistically significant positive correlation between PF_CK 18 and PF_Fetuin A (*r* = 0.096, *p* < 0.001), suggesting a synchronous increase in their levels in the PF. Conversely, PF_CK 18 demonstrates a statistically significant negative correlation with both plasma CK 18 (PLAS_CK 18, *r* = −0.173, *p* < 0.001) and PLAS_Fetuin A (*r* = −0.051, *p* < 0.001), indicating that higher levels of PF_CK 18 are associated with lower levels of these markers in the plasma. Furthermore, PLAS_CK 18 is positively correlated with PF_IL33 (*r* = 0.083, *p* < 0.001), PF_Fetuin A (*r* = 0.087, *p* < 0.01), and notably, PLAS_Fetuin A (*r* = 0.639, *p* < 0.001), which points to a strong association in plasma levels. An extraordinarily high correlation coefficient is observed between PLAS_CK 18 and PF_IL33_CT (*r* = 0.283, *p* < 0.01), suggesting a highly significant association that could indicate a robust interplay or co‐regulation in their biological functions.

These findings underscore the complex interdependencies between these biomarkers across different biological matrices. The strong positive correlation between PLAS_CK 18 and PLAS_Fetuin A particularly emphasises their potential co‐regulation in plasma and may suggest common pathways in their physiological or pathological roles. The high correlation between PLAS_CK 18 and PF_IL33_CT highlights the necessity for further investigation to elucidate the underlying mechanisms and implications of such a relationship. Understanding these correlations can provide deeper insights into the molecular mechanisms of diseases and potentially guide targeted therapeutic strategies.

To date, the studies have generally focused on serum concentrations for the systemic treatments of CAD. However, the components of PF reflecting the tissue environment have not been evaluated because the only possible way to obtain PF is invasive methods. Some studies have reported that some components in PF were changed in heart diseases [25, 26]. Component concentrations in PF and plasma could be different during the development of heart diseases. For instance, the pericardial concentrations of various angiogenic factors such as acid and basic fibroblast growth factors, vascular endothelial growth factors, and hepatocytic growth factors, which are known to stimulate collateral vessel development in ischemic heart diseases, are significantly increased [27, 28]. According to recent studies, the pericardium has become the application area of new treatment methods. Especially in patients in whom percutaneous intervention or surgical revascularisation cannot be performed, applications to stimulate collateral development in occluded vessels make the pericardium a new treatment intervention site and method [29, 30].

Studies have used troponin and natriuretic peptides as biomarkers to evaluate the pathogenesis, diagnosis, prognosis, and treatment of CAD. Although troponin is an important biomarker that plays a critical role in the regulation of cardiac muscle activity, cardiac troponins are not unique to CAD. They may increase in various conditions including acute respiratory distress syndrome, diabetes, acute neurological event, renal failure, chemotherapy, and drugs [31]. Natriuretic peptides are associated with many cardiovascular pathologies and are therefore used as biomarkers to evaluate the pathogenesis, diagnosis, prognosis, and treatment of CAD [32]. Levels of natriuretic peptides may also vary in relation to respiratory diseases, obesity, acute or chronic renal failure, and disorders in the endocrine system [33]. Therefore, it is important to know alternative biomarkers that change in relation to CAD. Within the scope of this study, the demonstration of alternative potential biomarkers for CAD in plasma and PF may provide a multidimensional perspective in the diagnosis and treatment phase in terms of the evaluation of many CAD‐related variables together.

**FIGURE 3:**
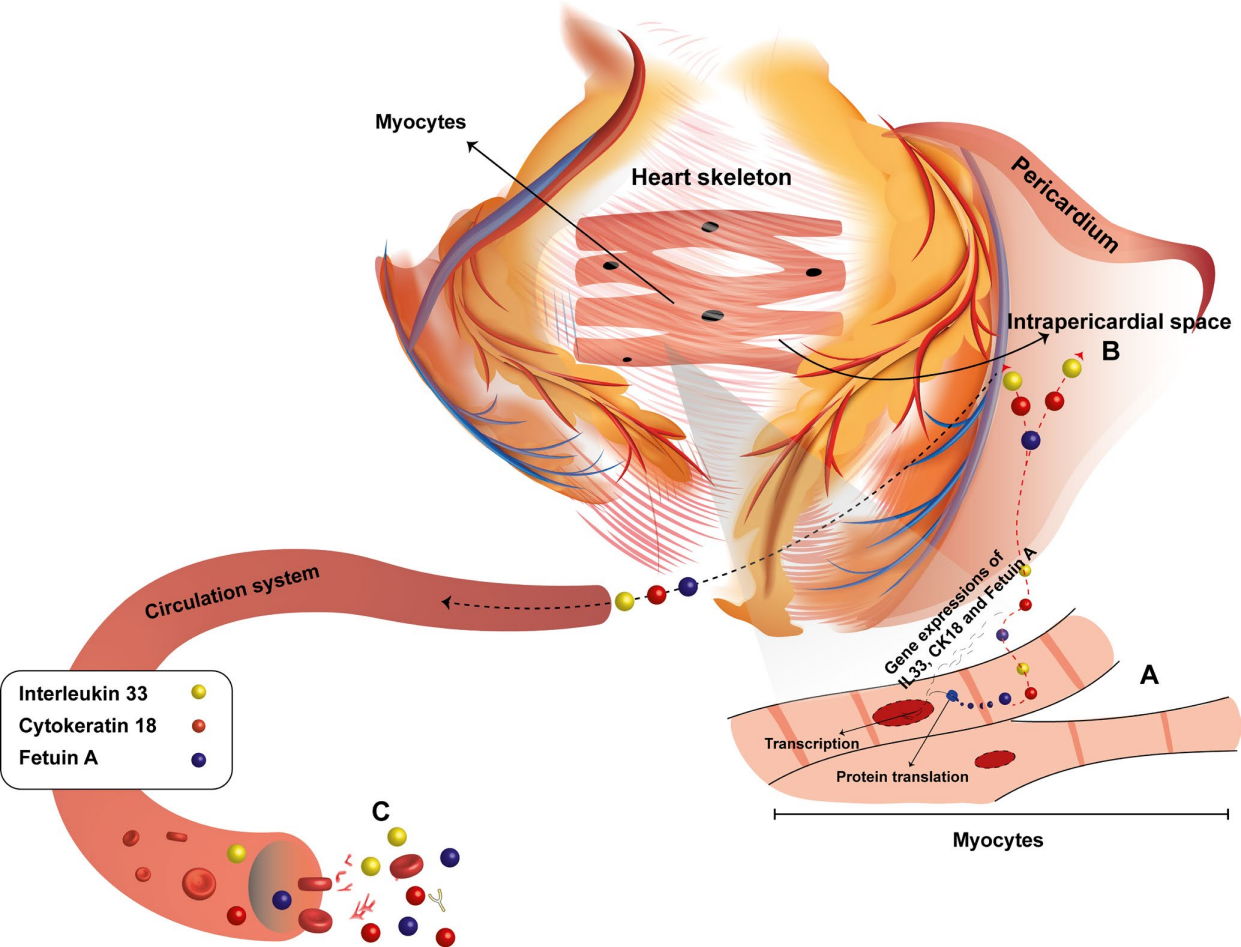
Gene expressions and protein levels of interleukin 33 and Fetuin A were found to be increased in PF compared to the plasma. The fact that while all organs contribute to IL 33, Fetuin A and CK 18 levels in plasma, the heart is the contributor to these biomarker levels in PF, suggest that measurement of these biomarkers in PF gives more valuable information about the heart.

Conclusion, It is obvious that IL 33, Fetuin A, and CK 18, limiting and repairing tissue damage, act as an important local link between metabolic disorders and physiological response. In our study, the significant changes of CAD‐related parameters such as IL 33, Fetuin A, and CK 18 genes and proteins in PF and plasma support the idea that these parameters can be used as a potential biomarker in heart diseases (Figure 3). Many treatment strategies can be developed based on the monitoring of IL 33, Fetuin A, and CK 18 levels in CAD or other cardiovascular diseases. PF is an important element that can be used for both diagnostic and therapeutic purposes in determining these strategies.

## 5 Limitations and Strengths

This study has several limitations. First, the sample size was relatively small (*n* = 40), which may limit the generalizability of the findings. Second, the study lacked a control group due to ethical constraints in obtaining pericardial fluid samples from healthy individuals. Third, the cross‐sectional design prevents establishing causal relationships between the measured biomarkers and CAD progression.

Despite these limitations, the study has notable strengths. It is among the few investigations to simultaneously assess both gene expression and protein levels of IL‐33, fetuin A, and CK‐18 in pericardial fluid and plasma of CAD patients. The use of pericardial fluid, which is in direct contact with cardiac tissue, provides valuable insights into localised cardiac‐specific pathophysiological changes. In addition, the combination of qRT‐PCR and ELISA techniques may offer significant contributions in terms of evaluating the results of molecular and protein level analyses together.

## Author Contributions

**Reşat Dikme:** investigation (equal), methodology (equal), validation (equal), writing – original draft (equal), writing – review and editing (equal). **Mehmet Salih Aydın:** investigation (equal), supervision (equal), writing – review and editing (equal). **Ebru Temiz:** investigation (equal), methodology (equal), writing – original draft (equal). **İsmail Koyuncu:** methodology (equal), writing – original draft (equal). **Mesut Işık:** investigation (equal), writing – original draft (equal), writing – review and editing (equal).

## Conflicts of Interest

The authors declare no conflicts of interest.

## Supporting information


Table S1.

## Data Availability

The data presented in this study is comprehensively shown in this article and its Supporting Information file. The underlying unprocessed raw data is available upon request.
