# Correction to ‘Potential Inflammatory Mediators in Pericardial Fluids of Patients With Coronary Artery Diseases and Their Association With Plasma Biomarkers’

**DOI:** 10.1111/jcmm.71319

**Published:** 2026-08-12

Dikme R, Aydın MS, Temiz E, Koyuncu İ, Işık M. “ Potential Inflammatory Mediators in Pericardial Fluids of Patients With Coronary Artery Diseases and Their Association With Plasma Biomarkers,” *Journal of Cellular and Molecular Medicine*, 2025; 29: e70625. https://doi.org/10.1111/jcmm.70625.

In the originally published version of this article, the Funding statement was incorrectly stated as follows:

‘The authors received no specific funding for this work’.

The correct Funding statement is:

‘This study was supported by Harran University Scientific Research Projects Coordination Unit (Project No. 17009)’.

The authors apologize for this error.

