# Supplementary material for: Potential Inflammatory Mediators in Pericardial Fluids of Patients With Coronary Artery Diseases and Their Association With Plasma Biomarkers
Source: J Cell Mol Med. 2025 May 28;29(11):e70625. doi: 10.1111/jcmm.70625 (PMC12119239; doi:10.1111/jcmm.70625)
Supplement: Supplementary file 1 — Table S1. [file JCMM-29-e70625-s001.docx]

**Table S1.** Comparisons of the biomarkers between PF nad Plasma

|  | Group | Mean | Std. Deviation | d | t | p |
| --- | --- | --- | --- | --- | --- | --- |
| CK18 ng/ml | PF | 1046.4278 | 431.36590 | 0.048 | 0.176 | 0.430 |
|  | Plasma | 1025.3181 | 448.55823 |  |  |  |
| IL33 ng/L | PF | 57.0963 | 10.80062 | 0.643 | 2.362 | 0.011 |
|  | Plasma | 50.1556 | 10.79274 |  |  |  |
| Fetuin A mg/L | PF | 1060.5324 | 481.44455 | 0.607 | 2.262 | 0.014 |
|  | Plasma | 725.8565 | 599.42095 |  |  |  |
| IL33 ∆ct | PF | 8.3033 | 3.09818 | -0.975 | -10.931 | 0.001 |
|  | Plasma | 18.2541 | 3.57408 |  |  |  |
| Fetuin A ∆ct | PF | 14.5519 | 0.48366 | -0.823 | -8.387 | 0.001 |
|  | Plasma | 21.0222 | 3.97958 |  |  |  |
